# Editorial: Cellular metabolism, the immune system, and oncogenesis: opportunities for drug discovery and development

**DOI:** 10.3389/fimmu.2026.1997905

**Published:** 2026-09-23

**Authors:** Monde Ntwasa, Ekene Emmanuel Nweke, Mandeep Kaur, Selisha Sooklal

**Affiliations:** 1 Department of Life and Consumer Sciences, College of Agriculture and Environmental Sciences, University of South Africa, Florida, Roodepoort, South Africa; 2 Department of Surgery, School of Clinical Medicine, Faculty of Health Sciences, University of the Witwatersrand, Johannesburg, South Africa; 3 Infectious Disease and Oncology Research Institute, Faculty of Health Sciences, University of Witwatersrand, Johannesburg, South Africa; 4 School of Molecular and Cell Biology, University of the Witwatersrand, Johannesburg, South Africa

**Keywords:** cancer, drug repurposing, immune checkpoint inhibitors, metabolic reprograming, tumour microenvironment

Nearly a century after Otto Warburg first described a unique phenomenon whereby tumour cells prefer the inefficient glycolytic pathway for energy generation (1), the phenomenon has continued to aid researchers in exploring the tumour ecosystem, including tumour initiation, progression and targeting. Furthermore, the Warburg effect has enabled researchers to interrogate how tumors and surrounding cells, such as immune and stromal cells, interact through shared and highly regulated metabolic processes. Tumor cells reprogram key metabolic processes, including glycolysis, lipid and amino acid processing, and nucleotide metabolism, to generate a “burst” of energy that fosters uncontrolled cellular proliferation (2). Deregulation of immune responses and metabolic processes has been recognized as a key hallmark of cancer (3).

Similarly, immune cells have been observed to exhibit similar processes. For example, a T-cell mounting an effector response against a foreign body requires a similar energy “burst”. In addition, a regulatory T-cell would prefer to use the oxidative phosphorylation pathway (4). Therefore, the intricate tumor microenvironment has been described as a battleground with ongoing competition for metabolic resources. In this context, cancer cells often win this competition, starving immune cells of critical nutrients such as glucose and creating a lactate-acidified cellular environment that incapacitates the immune system (5). The goal is not simply to inhibit a tumor-intrinsic pathway, but to shift a competitive balance so that immune cells regain access to the resources they need to survive and function effectively. Consequently, immune cells usually undergo deregulated metabolism before immunologic deficiency.

Cancer immunotherapy rests on the premise that the immune system can sufficiently recognize and kill tumor cells. Immunotherapeutic treatment must remove any impediment to this. For instance, checkpoint inhibitors work by releasing T cells and natural killer (NK) cells from inhibitory signals such as PD-1/PD-L1 and CTLA-4. Yet even where these therapies have improved outcomes, most patients with certain solid tumors still do not respond (6). Across cancer types, objective response rates to checkpoint blockade typically fall between 15% and 60%, with most solid tumors at the lower end and only melanoma and microsatellite-instability-high cancers approaching the upper end (7). A critical reason for this uneven outcome may be deregulated metabolism, reinforcing the relevance for drug discovery at the intersection of metabolism and immunity. One example showed that boosting a tumor’s own glycolysis is enough to override T cell control, but checkpoint blockade (anti-CTLA-4/PD-1/PD-L1) reverses this by restricting tumor glucose uptake, partly via PD-L1’s regulation of mTOR and glycolytic enzymes. As a result, glucose for T cells is freed up to fuel their glycolysis and IFN-γ production, showing that tumor-driven metabolic competition is a key mechanism of T cell dysfunction in cancer (8).

Over the past several years, researchers have made concerted efforts to decipher the crucial links between the immune response and metabolic processes. One of these links, called the lactylation axis, was comprehensively described in the present collection, where the authors explained a lactate-mediated mechanistic bridge that enables post-translational modification of immune signaling proteins (Tao et al.). It was also demonstrated that in hepatocellular carcinoma, the lactate transporter MCT4 actively drives matrix metalloproteinase activation and polarizes tumor-associated macrophages toward an immunosuppressive M2 phenotype (Zhang et al.). These studies describe metabolism not as a downstream consequence of oncogenic mutation but as a targetable node, echoing several calls in published literature to move from descriptive immuno-onco-metabolism toward precision targeting (9).

The papers in this collection span a wide range of malignancies and cover several metabolic pathways. In a study of acute myeloid leukemia, the authors showed that integrating transcriptomic data revealed substantial lipid metabolic heterogeneity across patient subgroups and highlighted GSTO1 as a potential therapeutic target within that heterogeneity (Zhong et al.). Another multi-omics analysis of ST3GAL4-mediated glycosphingolipid metabolism in triple-negative breast cancer linked this glycan-remodelling pathway to immune evasion and poorer prognosis (Zhang et al.). Researchers also investigated glucose metabolism, and one study showed that bile acid enrichment fuels aerobic glycolysis and immune evasion by stabilizing an FXR-RARα complex (Jiang et al.).

The breadth of the collection’s scope, spanning glucose, amino acid, lipid, and adenosine metabolism, also reflects that there is no single master metabolic switch to flip in targeting cancer cells. For example, lipid metabolism now shapes dendritic cell function, macrophage polarization, and the exhaustion trajectory of tumor-infiltrating lymphocytes (10, 11). Additionally, amino acid depletion, through arginase, tryptophan catabolism, and related pathways, remains one of the most direct mechanisms by which tumors disable adaptive immunity (12, 13).

Metabolic enzymes such as hexokinase, glutaminase, lactate dehydrogenase, and IDO are tractable drug targets, with established pharmacological inhibitors used to manage diseases such as diabetes, cardiovascular disease, and inflammatory diseases (14). Because tumor and immune metabolism are entangled, drugs that shift this metabolic balance can also improve the effectiveness of already approved immunotherapies, especially in previously non-responding patients, offering a lower-risk path to the clinic than *de novo* drug discovery (15). This has allowed metabolic pathway inhibitors to reach clinical testing faster than many oncogene-directed strategies. This collection offers instances of repurposing metabolic inhibitors for cancer. For example, Celecoxib, a drug developed for inflammation, was tested to prevent the malignant progression of smoking-induced lung tumors by suppressing COX-2/PGE2 signaling, a pathway associated with inflammation and metabolism (Sakurai et al.). In another study, Ailanthone, a natural compound, was found to induce apoptosis in osteosarcoma cells via endoplasmic reticulum stress, a mechanism increasingly recognized as intertwined with the lipid and protein-folding burdens carried by metabolically stressed tumor cells (Zhang et al.). However, metabolic pathways are shared. Consequently, a drug that starves a tumor of glutamine may just as easily starve the cytotoxic T cells sent to kill it (8). Therefore, a delicate balance must be maintained to ensure that immune cells still have sufficient energy to carry out their functions. Future research must focus on therapies that achieve this balance, ideally alongside existing immunotherapies, a goal this collection suggests is increasingly within reach.

## Conclusion

This collection of primarily original research articles showcases how metabolic rewiring in cancer cells and the tumor microenvironment drives immune evasion, therapy resistance, and oncogenesis, while identifying novel metabolic and immunological targets for drug discovery. Key findings span lipid metabolism in leukemia, bile acid-driven glycolysis, lactylation-mediated immunosuppression, and machine learning-based biomarker discovery. Critical thematic areas include metabolic drivers, immune modulation, lipid pathways, biomarkers, and Drug Repurposing. These thematic areas offer opportunities to target metabolic enzymes and receptors, reverse immunosuppression by exploiting post-translational modifications such as lactylation, discover lipid metabolism inhibitors and diagnostic/prognostic tools, and repurpose agents in oncology, such as Celecoxib. Overall, these articles underscore that metabolic rewiring is not just a hallmark of cancer cells but a systemic driver of immune dysfunction, opening multiple avenues for drug development, including metabolic enzyme inhibitors and immunometabolism combination therapies.

## References

[1] Warburg O . On the origin of cancer cells. Science. (1956) 123:309–14. doi: 10.1126/science.123.3191.309 13298683

[2] DeBerardinis RJ Chandel NS . Fundamentals of cancer metabolism. Sci Adv. (2016) 2:e1600200. doi: 10.1126/sciadv.1600200 27386546 PMC4928883

[3] Hanahan D . Hallmarks of cancer: New dimensions. Cancer Discov. (2022) 12:31–46. doi: 10.1158/2159-8290.CD-21-1059 35022204

[4] Shi Y Zhang H Miao C . Metabolic reprogram and T cell differentiation in inflammation: current evidence and future perspectives. Cell Death Discov. (2025) 11:123. doi: 10.1038/s41420-025-02403-1 40155378 PMC11953409

[5] Xia L Oyang L Lin J Tan S Han Y Wu N . The cancer metabolic reprogramming and immune response. Mol Cancer. (2021) 20:28. doi: 10.1186/s12943-021-01316-8 33546704 PMC7863491

[6] van Diepen AE Kucukcelebi S de Vos-Geelen J Kooreman NG . Immunotherapy for solid tumours: Current clinical landscape and future directions. Eur Surg Res. (2025) 67:21–9. doi: 10.1159/000549862 41379722 PMC12807497

[7] Das S Johnson DB . Immune-related adverse events and anti-tumor efficacy of immune checkpoint inhibitors. J Immunother Cancer. (2019) 7:306. doi: 10.1186/s40425-019-0805-8 31730012 PMC6858629

[8] Chang C-H Qiu J O’Sullivan D Buck MD Noguchi T Curtis JD . Metabolic competition in the tumor microenvironment is a driver of cancer progression. Cell. (2015) 162:1229–41. doi: 10.1016/j.cell.2015.08.016 26321679 PMC4864363

[9] Pajai S John JE Tripathi SC . Targeting immuno-oncology metabolism for precision cancer therapy. Front Oncol. (2023) 13. doi: 10.3389/fonc.2023.1124715 36816957 PMC9932929

[10] Herber DL Cao W Nefedova Y Novitskiy SV Nagaraj S Tyurin VA . Lipid accumulation and dendritic cell dysfunction in cancer. Nat Med. (2010) 16:880–6. doi: 10.1038/nm.2172 20622859 PMC2917488

[11] Ma X Bi E Lu Y Su P Huang C Liu L . Cholesterol induces CD8+ T cell exhaustion in the tumor microenvironment. Cell Metab. (2019) 30:143–56.e5. doi: 10.1016/j.cmet.2019.04.002 31031094 PMC7061417

[12] Rodriguez PC Quiceno DG Zabaleta J Ortiz B Zea AH Piazuelo MB . Arginase I production in the tumor microenvironment by mature myeloid cells inhibits T-cell receptor expression and antigen-specific T-cell responses. Cancer Res. (2004) 64:5839–49. doi: 10.1158/0008-5472.CAN-04-0465 15313928

[13] Uyttenhove C Pilotte L Théate I Stroobant V Colau D Parmentier N . Evidence for a tumoral immune resistance mechanism based on tryptophan degradation by indoleamine 2,3-dioxygenase. Nat Med. (2003) 9:1269–74. doi: 10.1038/nm934 14502282

[14] Hu T Liu C-H Lei M Zeng Q Li L Tang H . Metabolic regulation of the immune system in health and diseases: mechanisms and interventions. Signal Transduct Target Ther. (2024) 9:268. doi: 10.1038/s41392-024-01954-6 39379377 PMC11461632

[15] Pillai U J Ray A Maan M Dutta M . Repurposing drugs targeting metabolic diseases for cancer therapeutics. Drug Discov Today. (2023) 28:103684. doi: 10.1016/j.drudis.2023.103684 37379903

